# Spatial distribution of functional components in the starchy endosperm of wheat grains^⋆^

**DOI:** 10.1016/j.jcs.2019.102869

**Published:** 2020-01

**Authors:** Peter R. Shewry, Yongfang Wan, Malcolm J. Hawkesford, Paola Tosi

**Affiliations:** a Plant Science Department, Rothamsted Research, Harpenden, Herts, AL5 2JQ, UK; b School of Agriculture, Policy and Development, University of Reading, Whiteknights Campus, Early Gate, RG6 6AR, Reading, UK

**Keywords:** Wheat, White flour, Starchy endosperm, Starch, Lipids, Gluten proteins, Polysaccharides, Dietary fibre, A, arabinose, AX, arabinoxylan, AXOS, arabinoxylan oligosaccharide, DP, degree of polymerisation, DPA, days past anthesis, FTIR, Fourier transform infrared, GL, galactolipid, HMW, high molecular weight, LMW, low molecular weight, NMR, nuclear magnetic resonance, TAG, triacylglycerol, PL, phospholipid, SIMS, secondary ion mass spectrometry, TDF, total dietary fibre, WE, water-extractable, WU, water-unextractable, X, xylose

## Abstract

The starchy endosperm of the mature wheat grain comprises three major cell types, namely sub-aleurone cells, prismatic cells and central cells, which differ in their contents of functional components: gluten proteins, starch, cell wall polysaccharides (dietary fibre) and lipids. Gradients are established during grain development but may be modified during grain maturation and are affected by plant nutrition, particularly nitrogen application, and environmental factors. Although the molecular controls of their formation are unknown, the high content of protein and low content of starch of sub-aleurone cells, compared to the other starchy endosperm cells types, may result from differences in developmental programming related to the cells having a separate origin (from anticlinal division of the aleurone cells). The gradients within the grain may be reflected in differences in the compositions of mill streams, particularly those streams enriched in the central and outer cells of the starchy endosperm, respectively, allowing the production of specialist flours for specific end uses.

## Highlights

• The mature starchy endosperm of wheat comprises three cell types.
• These differ in their contents of functional components.
• These differences are reflected in the compositions of mill streams.
• These differences may affect functionality.
• Hence innovative milling can be used to prepare flours for special uses.

## Graphical abstract

**Figure:**
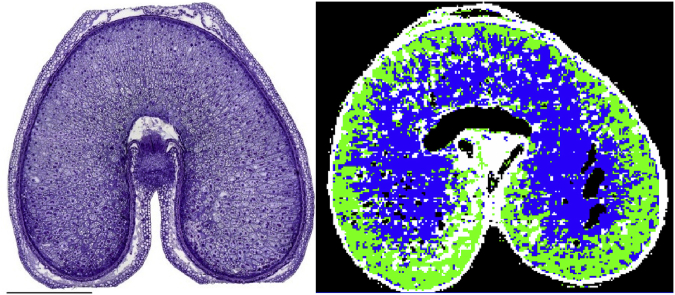

## 1 Introduction

Wheat is used for a wide range of foods, from globally consumed forms such as bread, cakes, biscuits, pasta and noodles to regional and traditional foods such as couscous and bulgar. As the mature wheat kernel is dry and hard it can only be consumed after processing, which for most products starts with a form of milling. Milling fulfils two purposes: to reduce the grain to flour and to separate the different grain tissues. This separation is required because the grain has a complex structure, comprising several tissues which differ in their compositions, functional properties, health benefits and palatability to consumers. For most food applications milling is used to separate the starchy endosperm tissue of the grain and reduce it to fine white flour. However, this coarse separation ignores the fine differences in composition which exist both within and between tissues. We consider that this variation can be exploited by innovative milling methods, to develop specialist flours for specific end uses.

### 1.1 The wheat grain

The wheat grain is a single seeded fruit (caryopsis) in which the maternal fruit coat (pericarp) and seed coat (testa) surround the zygotic embryo and endosperm (Bechtel et al., 2009). These tissues can be further divided: the embryo comprises a single storage cotyledon (the scutellum) and the embryonic axis (plumule, radicle and hypocotyl) while the endosperm consists of starchy endosperm cells surrounded by a single layer of aleurone cells. The major tissue in the mature grain is the starchy endosperm, which accounts for about 83–84% of the dry weight. By contrast, the embryo accounts for about 3% of the dry weight, the aleurone about 6.5% and the outer layers (pericarp and testa) about 7–8% (Barron et al., 2007).

The starchy endosperm is the major storage tissue, storing both starch and protein, and is the origin of white flour produced by milling. Millers aim to recover the highest possible proportion of this tissue, often achieving white flour yields equivalent to 78–80% of the grain dry weight. However, this process is based on the assumption that the endosperm is essentially homogeneous in composition which is not the case.

**Fig. 1:**
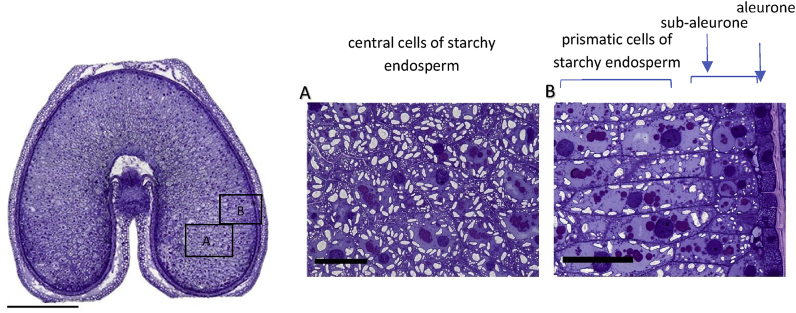
Cross section of a developing grain of durum wheat cv Ofanto at 20 days DPA, stained with toluidine blue to show the distribution of protein (taken from Tosi et al., 2009, 2018). The left hand image shows the whole grain with the areas in boxes A and B expanded in the central and right hand images, respectively. The bar in the cross-section represents 1 mm, the bars in panels 1 and 2 100 μm. Note the high concentration of protein in the sub-aleurone cells in area B. (For interpretation of the references to colour in this figure legend, the reader is referred to the Web version of this article.)

In fact, studies of developing and mature grain show that the cells present in the starchy endosperm differentiate into three major types (which are illustrated in Fig. 1) (Evers and Millar, 2002). Beneath the single layer of aleurone cells are two to three layers of protein-rich sub-aleurone cells and beneath these are elongated prismatic cells which radiate towards the centre of the grain (Fig. 1, area B). Finally, the cells in the central parts of the cheeks of the grain are round or polygonal and become highly extended with starch (Fig. 1, area A). Bradbury et al. (1956) reported approximate sizes of 60 μm diameter for the sub-aleurone cells, 128–200 μm x 40–60 μm for the prismatic cells and 72–144 × 69–120 μm for the central cells.

These differences in cell size and morphology are accompanied by differences in composition, resulting in radial and longitudinal gradients.

### 1.2 Analysis of gradients by pearling and imaging

Two approaches have been taken to study gradients in developing and mature grain. Sections of grain tissue can be analysed by various imaging approaches, ranging from simple light microscopy of stained and fixed tissues with components visualised by staining or immunochemistry, to sophisticated chemical imaging such as Fourier Transform Infrared (FT-IR) microspectroscopy (Barron et al., 2005; Toole et al., 2009, 2010, 2011), Raman microspectroscopy (Phillipe et al., 2006; Toole et al., 2009), MALDI MSI (Fanuel et al., 2018), and Secondary Ion Mass Spectrometry (NanoSIMS) (Moore et al., 2016). In general, these approaches are more readily applied to developing tissues than to mature grain, due to the ease of sectioning and the lower content of starch. Chemical imaging also requires specialist equipment and is generally low throughput. For example, modern NanoSIMS equipment costs in excess of £3m with a maximum throughput of about 1 sample a day.

**Fig. 2:**
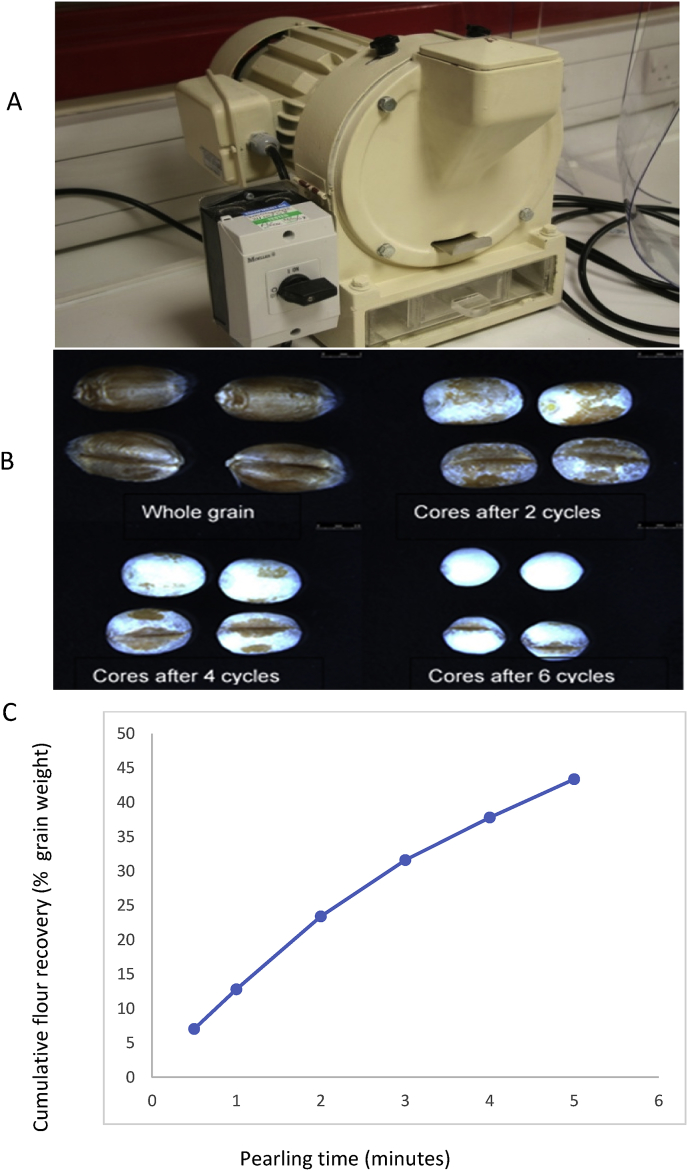
Pearling of grain of wheat cv Hereward. Panel A shows the pearling mill; panel B the whole grain and the cores after a typical experiment of 6 pearling cycles; panel C the cumulative removal of material from the grain over 6 cycles using a single grain sample. Based on He et al. (2013)

Although it is possible to carry out biochemical and chemical analyses on material prepared by hand-dissection of grain (for example, Saulnier et al., 2009), this approach is limited by the amount of time required for preparation. A more widely used method is to remove sequential fractions from the outside of the grain using pearling (as described for barley by Millet et al., 1991 and for wheat by He et al., 2013). The application of pearling to wheat is illustrated in Fig. 2. Laboratory scale pearling mills, such as that shown in Fig. 2, are generally used for grain samples of about 50 g, to generate fractions of between 3 and 5 g each. However, these fractions do not correspond precisely to botanical tissues and pearling has two important limitations. Firstly, because the grain is elongated the removal of tissue is not even, with more removed from the end of the grain (which consequently becomes progressively more rounded). Secondly, the tissue within the groove is not removed, with the groove still being apparent when over 40% of the grain weight has been removed (Fig. 2). Nevertheless, the fractions still provide a broad view of the distribution of components within the whole grain.

## 2 Radial gradients in the starchy endosperm

### 2.1 Protein

Differences in protein content and composition have been known for many years, with the sub-aleurone cells being richer in protein and having fewer and less regular in shape starch granules, compared with other starchy endosperm cells (Bradbury et al., 1956; Kent, 1966; Kent and Evers, 1969). In fact, Kent (1966) calculated that the sub-aleurone cells in a flour of 12.5% protein contained 54% protein (whereas 8% would be more typical for the central cells), and this protein enrichment of the sub-aleurone can be clearly seen when grain sections are stained to show the distribution of protein and other components (Fig. 1).

In addition, there are well established gradients in protein composition. The most detailed study so far used sequential pearling to remove six fractions each corresponding, on average, to about 8% of the grain weight (He et al., 2013). Comparison of these fractions and the milled core (corresponding to about 50% of the grain weight) showed that although the total protein content decreased from the outer layers to the centre of the grain, the proportion of gluten proteins increased, from about 50 to 55% to about 75% of the total protein in grain from plants grown with 100 kg/ha of N-fertilizer. Analysis of protein fractions by SDS-PAGE and western blotting with antibodies to gluten proteins shows that there are also gradients in gluten protein composition, with the proportions of HMW subunits of glutenin and ɣ-gliadins increasing toward the centre of the grain and proportions of some Ѡ-gliadins and α-gliadins decreasing (Tosi et al., 2011; He et al., 2013). However, SDS-PAGE does not clearly separate the LMW subunits of glutenin from ɣ-gliadins and α-gliadins. He et al. (2013) therefore also determined the size distribution of gluten proteins extracted using SDS by SE-HPLC (Morel et al., 2000). This showed clear increases, from the outside to the centre of the grain, in the proportions of the HMW glutenin polymers (%F1) that are considered to contribute to gluten elasticity (Shewry et al., 2003) and, to a lesser extent, in the lower molecular weight glutenin polymers (%F2). These gradients were reflected in an increase in the ratios of HMW:LMW polymers (%F1/%F2 ratio), and of glutenin (F1+F2) to gliadin (F3 comprising mainly Ѡ-gliadins + F4 comprising α- and ɣ-gliadins). Both of these ratios have been used as predictors of breadmaking quality.

Zhou et al. (2018) also reported analyses of pearling fractions, but removed 8 fractions, each corresponding to about 10% of the grain weight, with the core representing only 20% of the total. The results were broadly consistent with those of He et al. (2013), although they determined glutenin macropolymers (GMP), rather than the size distribution of glutenin polymers by SE-HPLC, and expressed the amounts of all proteins as % dry weight.

### 2.2 Starch

Starch is a mixture of two glucose polymers: amylose, which consists of unbranched (1 → 4) α-linked chains comprising up to several thousand glucose units, and amylopectin, which may comprise over 100,000 glucose units and is highly branched with (1 → 6) α-linkages as well as (1 → 4) α-linkages. The proportion of amylose in wheat starch generally ranges from about 18% to 35%. Mature wheat grain contains two distinct populations of starch granule, referred to as A-type and B-type. These populations differ in size and morphology, with A-type being >10 μm and lenticular in shape and B-type <10 μm and spherical in shape (Stone and Morell, 2009). These populations also differ in polymer composition and structure (Shinde et al 2003), with B-type granules containing lower proportions of amylose than A-type granules (Duffus and Murdoch, 1979; Shinde et al., 2003).

Microscopy of developing and mature grain shows clear differences in the distribution of starch within the starchy endosperm cells, with only a few small granules being present in the protein-rich sub-aleurone cells (Fig. 1) (as also discussed by Tomlinson and Denyer, 2003). This distribution is consistent with the increases in the total starch and % amylose reported in pearling fractions by Tosi et al. (2018) and Zhou et al. (2018).

More detailed studies were reported by Zhou et al. (2018), who classified the starch granules into three types: A (diameter 22–28 μm), B (7.4–7.7 μm) and C (2.9–3.24 μm) and showed small but statistically significant differences in their mean diameters between pearling fractions.

Starch has a major impact on the processing properties of flours, and both Tosi et al. (2018) and Zhou et al. (2018) reported gradients in the properties of the starch (onset temperature of gelatinisation and pasting properties) present in pearling fractions. However, it should be noted that these properties are likely to be affected by effects of milling, particularly on starch damage, which may be greater using a pearling mill (up to 18% damaged starch being reported by Tosi et al., 2018) than in roller milling (generally up to about 12%).

### 2.3 Cell wall polysaccharides

Cell wall polysaccharides are the major source of dietary fibre in cereal products and hence are important for human nutrition and health. Whole grain contains about 11.5–15.5% (mean 13.4%) total dietary fibre (TDF), including 5.53–7.42% (mean 6.49%) arabinoxylan (AX), 1.67–3.05% (mean 2.11%) cellulose and 0.51–0.96% (mean 0.73%) β-glucan (Andersson et al., 2013). However, all of the cellulose and much of the AX and β-glucan are located in the outer (bran) layers, and white flour contains only about 2–3% cell wall polysaccharides, with AX (70%) and β-glucan (20%) being the dominant components. Analyses of pearling fractions have shown that TDF, AX and β-glucan all decrease in concentration from the outer to inner layers (Zhou et al., 2018; Tosi et al., 2018). However, a series of studies have focused on variation in the fine structures of these components.

AX comprises a backbone of β-D-xylopyranosyl (xylose) residues linked through (1 → 4) glycosidic linkages, with some residues being substituted with α-L-arabinofuranosyl (arabinose) residues at either one or two positions. Some arabinose residues present as single substitutions may be further substituted with ferulic acid at the 5-position, which may form diferulate cross-links between polymers. AX is often divided into two classes, depending on whether it is extractable (WE-AX) or unextractable (WU-AX) with water. β-glucan comprises glucose residues joined by (1 → 3) and (1 → 4) linkages. Single (1 → 3) linkages are usually separated by two or three (1 → 4) linkages, but longer stretches of up to 14 (1 → 4) linked glucan units (sometimes referred to as “cellulose-like” regions) have been reported for wheat bran β-glucan (Li et al., 2006). This structural variation may be studied by “enzyme fingerprinting” (Ordaz-Ortiz et al., 2005), in which the polymers are digested with specific enzymes (endoxylanase for AX, lichenase for β-glucan) and the structures and proportions of the oligosaccharides which are released determined, by spectroscopic imaging of sections, or by NMR spectrometry of hand dissected samples of tissue.

**Fig. 3:**
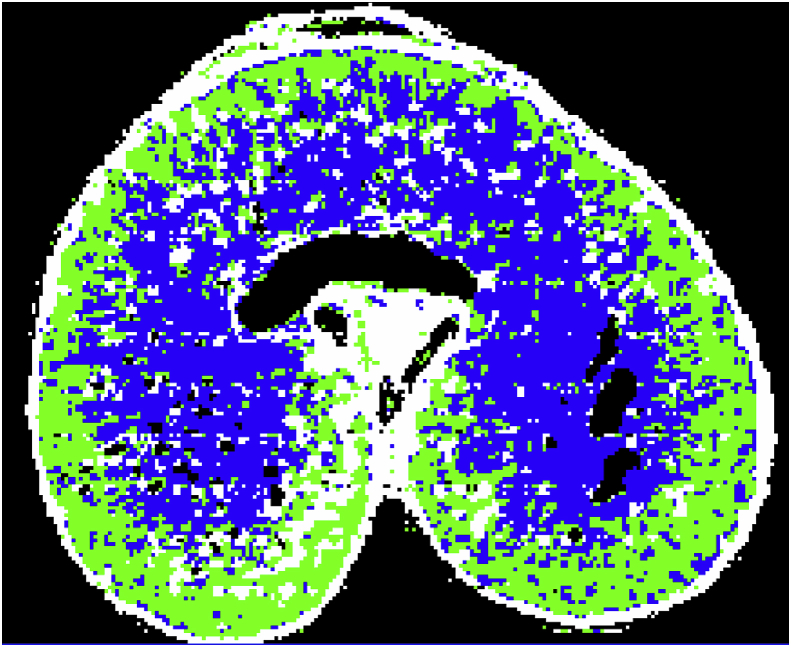
Spectroscopic FT-IR image overlaid onto a light microscope image of a transverse section of a grain of wheat cv. Spark at 30 DPA. The grain section has been treated to remove the cell contents allowing the spectra of the cell walls to be determined. Previous studies had established that the height of a shoulder in the FT-IR spectrum at 1075 cm-1 reflects the extent of substitution of the AX structure (Toole et al., 2007, 2009). A colour was therefore assigned to each pixel depending on the height of the shoulder at 1075 cm-1 compared to that of the major peak at 1041 cm-1. If the shoulder was below 66% the pixel was coloured green to represent low substituted AX and if it was above 66% it was coloured blue to represent highly substituted AX. White represents remaining starch, and black represents holes or pixels where the amount of AX was too low to determine. Figure kindly provided by Dr. Geraldine Toole (IFR, Norwich, UK). (For interpretation of the references to colour in this figure legend, the reader is referred to the Web version of this article.)

Barron et al. (2005) developed a protocol for comparing the structure of AX in the cell walls of transverse sections of wheat grain, using sonication and washing with 70% ethanol to remove the cell contents (notably starch) and protein adhering to the cell wall, and then using FT-IR microspectroscopy to determine the structure of AX. Comparison of the spectra with those of purified WE-AX and WU-AX allowed AX structures to be defined as highly substituted with arabinose or less highly substituted. Fig. 3 illustrates this approach, using false colour to display the distributions of highly substituted AX (blue) in the centre of the grain and less substituted AX (green) towards the periphery. However, it should be noted these two structures were defined using an arbitrary cut off and are not discrete populations of molecules. Similarly, there are gradients rather than discrete boundaries in the distributions of this structural variation across the grain. Further application of this method showed that the relative degree of arabinosylation varied between cultivars grown under the same conditions (Toole et al., 2011), and that the structure changed during grain development, with a decrease in the area of highly substituted AX and an increase in the area of less substituted AX, a process referred to as “remodelling” (Toole et al., 2010).

FT-IR gives only limited information on AX structure and other approaches have been used to provide more detailed information on variation in structure at the tissue level, including Raman microspectroscopy which provides data on esterification with phenolic acids as well as arabinosylation (Phillipe et al., 2006; Toole et al., 2009), ^1^NMR spectrometry to provide more information on arabinosylation (Toole et al., 2010, 2011) and micro-scale enzyme fingerprinting (as discussed above) (Saulnier et al., 2009). These earlier studies have been reviewed in detail by Saulnier et al. (2009).

More recently, Saulnier and colleagues have carried out enzyme hydrolysis directly on tissue sections and identified the oligosaccharides released by MALDI mass spectroscopy imaging (MSI) (Velickovik et al., 2014). In order to study spatial variation in structure they determined four oligosaccharides: DP3 and DP4 fragments from β-glucan and AX5 and AX6 fragments from AX. The latter have the structures XA_3_XX and XA_2+3_XX and were selected as they are known to be major contributors to variation in AX substitution. They showed that both AX and β-glucan were concentrated in the outer cells of the endosperm in immature grain but more evenly distributed throughout the endosperm at maturity. The ratio of AX5/AX6 fragments also confirmed other studies (discussed above) which showed lower arabinosylation of AX in the outer layers. Similarly, the ratio of DP3/DP4 fragments released from β-glucan was higher in the glucan-enriched outer cells of immature grain.

### 2.4 Lipids

Lipids are minor components of the grain, accounting by weight for about 2.0–2.5% of flour (Pareyt et al., 2011). They comprise many individual components (molecular species) which are broadly classified into three types: polar lipids (phospholipids (PL) and galactolipids (GL) which are structural components of membranes), triacylglycerols (TAG) (storage lipids) and free fatty acids. However, there is great diversity within all three groups, notably in the head groups of PLs and GLs and the fatty acids esterified to these components and TAGs. The total lipid content of pearling fractions is greatest in those that contain the aleurone and embryo and the lowest in fractions corresponding to the centre of the grain, with the proportion of unsaturated fatty acids showing a similar pattern (Tosi et al., 2018). The composition of molecular species also varies between pearling fractions, including the contents of GLs (monogalactosyl diglyceride and digalactosyl diglyceride) (González-Thuillier et al., 2015).

## 3 Linear gradients in the starchy endosperm

Early reports of differences in the distribution of components in the wheat starchy endosperm focused on radial gradients as these are readily observed in transverse sections of grain. Consequently, analyses of pearling fractions were also largely interpreted in relation to radial distribution. However, pearling actually removes more material from the ends of the grains than from the central parts, resulting in an increasingly spherical shape (Fig. 1). Hence, the question must be asked whether gradients also exist along the longitudinal axis of the grain. Although there is little work on this topic, two recent studies show that this is the case.

### 3.1 Proteins

Shi et al. (2019) determined longitudinal gradients in proteins by removing the embryos from developing caryopses and then cutting them into three equal sections. The total gluten protein content was lower in the section adjacent to the embryo, which may have related to the presence in the dorsal part of modified starchy endosperm cells which support the growth of the embryo. However, gradients in protein composition were also observed, with a lower proportion of Ѡ-gliadins (and higher proportions of other gluten proteins) in the section adjacent to the embryo. The biological significance of this distribution is not known, as there is no obvious relationship between Ѡ-gliadins and embryo development.

### 3.2 Cell wall polysaccharides

Saulnier et al. (2009) reviewed the current evidence for variation in cell wall polysaccharides along the longitudinal axis of the grain. They reported that the starchy endosperm cells close to the embryo (proximal to the point of attachment) were enriched in β-glucan, with AX being highly substituted (with a ratio of A:X of about 0.7) in the same cells. The proportion of β-glucan decreased towards the distal end of the grain, with lower AX substitution, although the substitution was higher in prismatic cells than in central cells.

A more detailed study was reported by Fanuel et al. (2018), who analysed 30 consecutive cross-sections of a mature grain using enzyme digestion to release oligosaccharides from β-glucan and AX, which were then detected by MALDI MSI (as discussed above). Compilation of the images allowed a 3D model of variation in polysaccharide structure to be constructed. This confirmed that β-glucan was more abundant adjacent to the germ and in the central starchy endosperm cells, while the AX was more highly substituted at the distal end of the grain and around the crease.

Films made with highly substituted AX show higher water-diffusivity than those made with low substituted AX (Ying et al., 2015) and Saulnier et al. (2009) suggested that structural variation in AX may modulate the hydration properties of cell walls. Fanuel et al. (2018) therefore concluded that the distribution of highly substituted AX along the crease and particularly in the vicinity of the germ, was consistent with the active transport of nutrients in these regions.

## 4 Modulation of gradients by nutrition and environment

**Fig. 4:**
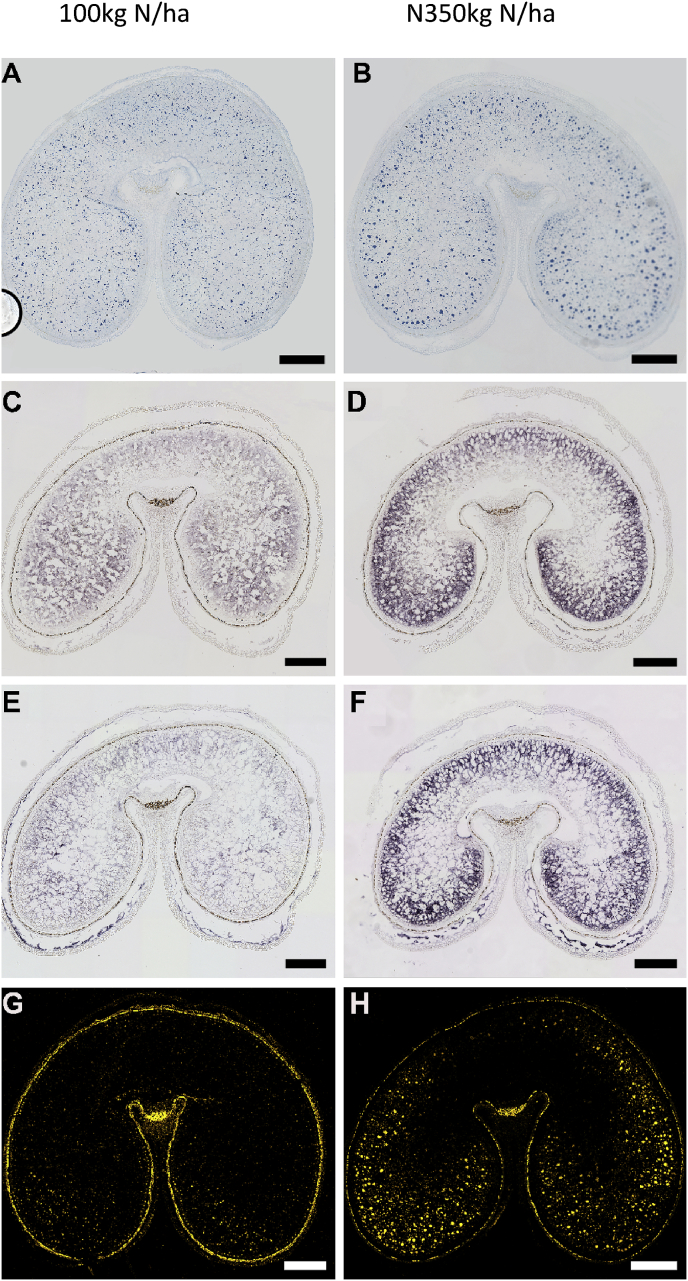
Spatial patterns of deposition of total proteins and ω-gliadins in the starchy endosperm of wheat cv Hereward grown at nitrogen levels of 100 kg/ha (left panel: A, C, E, G) and 350 kg/ha (right panel: B, D, F, H). A-B, sections at 27 DPA stained for protein bodies with Naphthol Blue Black; E-F, *in situ* hybridisation of transcripts related to ω2-gliadins (C,D) and ω5-gliadins (E,F) at 17 DPA; G-H, immunolocalisation of ω5-gliadin at 27 DPA; The immunofluorescence labelling in G and H is displayed in false yellow colour. Scale bars: 500 μm (A–H). Taken from Wan et al. (2013). (For interpretation of the references to colour in this figure legend, the reader is referred to the Web version of this article.)

He et al. (2013) compared the spatial patterns of gluten proteins and polymers in wheat grain grown with two levels of nitrogen fertiliser, 100 kg Ha (which is typical of low input farming systems in the UK) and 350 kg Ha (which is higher than used by UK farmers). Nitrogen availability had the expected positive effect on total grain protein and on total gluten proteins, but also affected the protein composition, with the proportions of Ѡ-gliadins increasing in all pearling fractions, and the proportion of HMW subunits increasing in all fractions except the core. This differential effect of nitrogen is illustrated in Fig. 4, which shows gradients in the amounts of Ѡ-gliadin protein and RNA transcripts in transverse sections of grain grown at 100 and 350 kgN Ha (Wan et al., 2013).

Similar differential effects of N-supply on gluten protein composition in three longitudinal sections were reported by Shi et al. (2019), with increased proportions of Ѡ-gliadins and HMW subunits in all three sections at 21 and 28 days post anthesis. However, He et al. (2013) reported that there was little effect of nitrogen supply on the proportions of glutenin polymers in the fractions, with the percentages of peaks F1 (comprising high molecular weight polymers enriched in HMW subunits of glutenin) and F2 (comprising smaller polymers enriched in LMW subunits of glutenin) being only marginally lower at 350 kg N Ha and the ratio of %F1/%F2 being almost identical in grain grown at both nitrogen levels.

More detailed studies of factors determining gradients in protein amount were reported by Savill et al. (2018) who developed an image analysis system to quantify the distribution of protein in stained sections of developing grain. In addition to confirming previous studies of protein distribution and the effects of nitrogen fertilisation, they also showed that the gradients were enhanced by high temperatures post-anthesis. Zhong et al. (2018) also studied the effects of nitrogen fertilisation on protein distribution and processing quality, showing that they could be modulated by the timing of application of top dressing.

Environmental effects on the remodelling of AX have also been reported, with the rate of transition from highly arabinosylated to less arabinosylated AX being faster in grain grown at higher temperature and limited water availability from 14 days after anthesis (Toole et al., 2007). However, it is likely that this acceleration results from the increased rate of grain maturation under these conditions rather than reprogramming of grain development.

## 5 Mechanisms determining the establishment of gradients during development of wheat grain

**Fig. 5:**
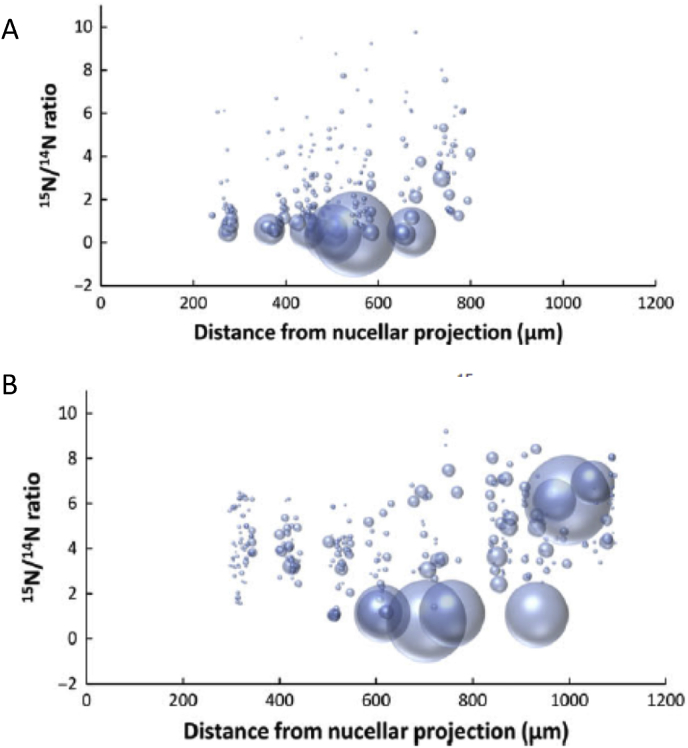
Graphical representation of the size and ^15^N enrichment of protein bodies along a transect of starchy endosperm tissue (from the nucellar projection to the aleurone layer) after labelling at 20 DPA and imaging after either 24 h (A) or 7 days (B), showing transport of ^15^N glutamine substrate across the developing starchy endosperm. Individual protein bodies are displayed as “bubbles”, which correspond in size to their measured areas. The positions of the protein bodies correspond to their locations along the transect (x axis) and their degree of enrichment with ^15^N (y axis). Sections were washed to remove free ^15^N glutamine Taken from Moore et al. (2016).

Microscopy of developing wheat grains using specific antibodies for immunolocalisation shows that the gradients in protein content and composition in the starchy endosperm are established gradually during development, with different proteins accumulating at different rates at different stages (Tosi et al., 2011). However, such analyses are only able to measure protein accumulation, not protein deposition at a defined stage. In order to study this, Moore et al. (2016) fed ^15^N-labelled glutamine to developing grains, via microcapillary tubes inserted into the rachis, and determined protein deposition by measuring the degree of enrichment of protein bodies in individual cells with ^15^N using NanoSIMS (secondary ion mass spectrometry). Isotope was fed for 6 h and the developing caryopses harvested either immediately, after 24 h or after 7 days. This showed that the labelled substrate was transported radially from its point of entry in the groove across the central starchy endosperm to the protein-rich sub-aleurone cells. This is illustrated in Fig. 5, which shows that after 7 days most of the ^15^N is present in large protein bodies in the sub-aleurone cells.

This raises the question of why the amino acid substrate is transported across the developing starchy endosperm to be incorporated into protein in the cells just below the aleurone layer. The simplest explanation is that the genes encoding gluten proteins are also differentially expressed, being most strongly expressed in the sub-aleurone cells. This is certainly the case for a LMW subunit gene, which is strongly expressed in these cells but only weakly expressed in the central starchy endosperm (Stoger et al., 2001). Thus, high levels of gluten protein gene expression may provide a sink for amino acid substrates in the sub-aleurone cells, resulting in a concentration gradient which drives transport from the transfer cells in the groove. This hypothesis is consistent with the study of Ugalde and Jenner (1990) who measured the concentrations of soluble amino acids across the developing endosperm. They found that the concentrations decreased from the endosperm cavity to the mid-point and then increased from the midpoint to the periphery. Based on this they concluded that the high level of protein accumulation in the peripheral cells could not be attributed to the pattern of substrate supply and that the transport of amino acids did not limit protein synthesis.

The difference in developmental programming of the sub-aleurone cells with respect to other starchy endosperm cells may result from their different lineages, the sub-aleurone cells being derived from anticlinal divisions of the aleurone cells, which continue to divide up to about 14 days after anthesis (Bechtel et al., 2003). This differential programming may also account for the differences in the accumulation of starch between the sub-aleurone and other starchy endosperm cells.

Whereas the gradients in protein composition may be explained, at least in part, by differences in developmental programming due to cell lineages, the gradients in other components are not as clear cut and the explanations may differ. In particular, Saulnier and colleagues have suggested that variation in AX structure modulates the hydration and permeability of cell walls and that variation in composition reflects the functional requirements of the cells. Hence, in this case the gradients may reflect biological rather than developmental differences between cell types.

## 6 Is it possible to exploit spatial gradients in grain utilisation?

The compositions of the fractions removed by pearling clearly reflect differences in the spatial distributions of components within the grain that could have impacts on processing quality. For example, the increased proportion of high molecular weight glutenin polymers in the central part of the grain reported by He et al. (2013) implies that the central cells of the starchy endosperm would have higher intrinsic quality for breadmaking, despite their lower protein content. Similarly, the high content in galactolipids of the central core (González-Thuillier et al., 2015) may affect the breadmaking performance (Pareyt et al., 2011). Differences in starch properties among pearling fractions (He et al., 2013; Zhou et al., 2018) would also be expected to affect processing quality, and Zhou et al. (2018) indeed reported correlations with differences in quality for making bread and biscuits.

The question, therefore, is whether these gradients can be exploited by conventional milling to produce flours with different compositions and end use properties. Milling is a highly sophisticated process which has been developed to separate the starchy endosperm tissue (white flour) from the outer layers (aleurone, pericarp and testa) and embryo (germ), which together form the bran. Laboratory scale mills may produce up to 6 flour fractions, and commercial mills over 20. In commercial milling, the purest flour fractions are recombined to give white flour, with a total yield of between 78% and 80% of the grain weight.

However, the flour fractions are known to differ in composition. This difference in purity may reflect their degree of contamination with bran. However, it may also reflect their origin in the grain, with the purest streams coming from the central part of the starchy endosperm. It is therefore of interest to determine how their compositions compare with those of pearling fractions, so that mill streams can be more efficiently recombined to produce flours with specific characteristics.

**Fig. 6:**
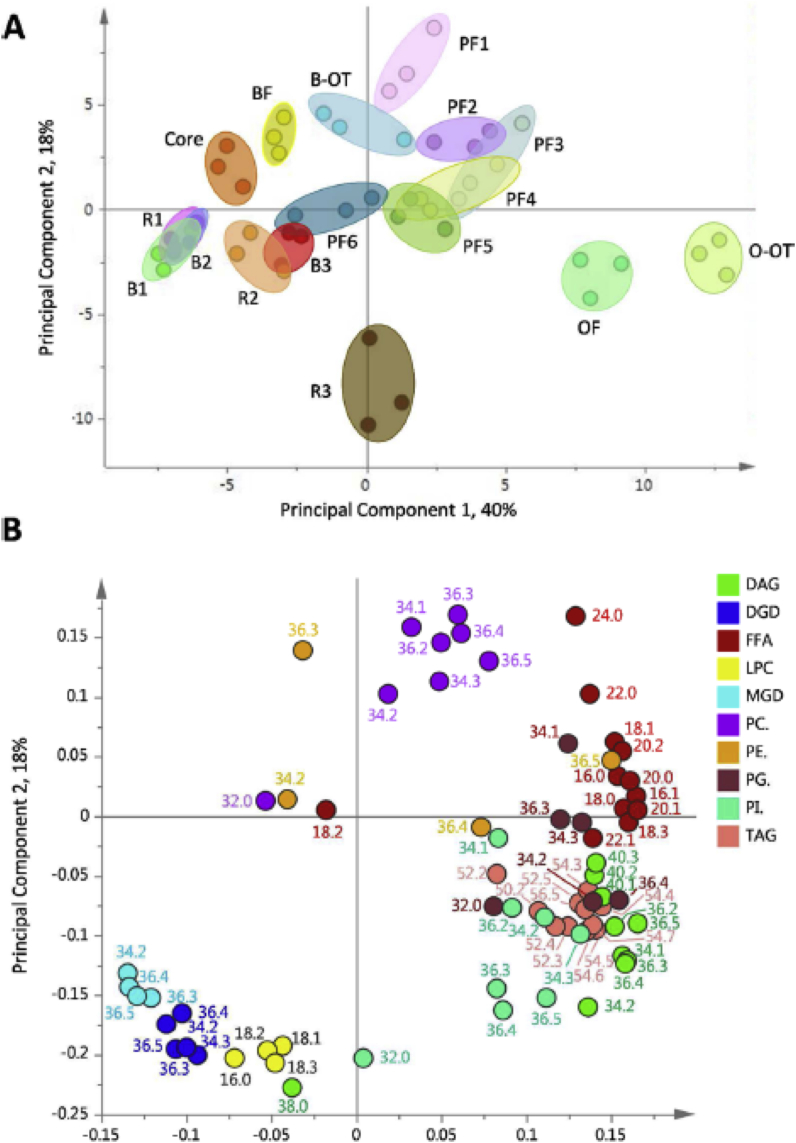
Principal Component Analysis (PCA) fractions from milling and pearling of wheat cv Hereward A: PCA scores plot showing PC1 (40%) vs PC2 (18%). Pearling fractions: PF1, pearling fraction 1; PF2, pearling fraction 2; PF3, pearling fraction 3; PF4, pearling fraction 4; PF5, pearling fraction 5; PF6, pearling fraction 6 and core. Milling fractions: B1, break 1; R1, reduction 1; B2, break 2; R2, reduction 2; B3, break 3; R3, break 3; OF, offal fraction; O-OT, offal over-tail; BF, bran fraction; B-OT, bran over-tail. B: PCA loadings plot of PC1 vs PC2 showing the molecular species responsible for the separation in A. Variables are coloured according to their lipid class and are labelled according to chain length and double bond number. Lipid classes are diacylglycerol (DAG), digalactosyl diglyceride (DGD), free fatty acids (FFA), lysophosphatidyl choline (LPC), monogalactosyl diglyceride (MGD), phosphatidyl choline (PC), phosphatidyl ethanolamine (PE), phosphatidyl glycerol (PG), phosphatidyl inositol (PI), and triacylglycerol (TAG)., Taken from González-Thuillier et al. (2015) with permission.

In order to answer this question González-Thuillier et al. (2015) compared the lipid compositions of pearling fractions with mill streams produced using a Buhler-MLU-202 laboratory mill, using the same grain sample. The mill generated 10 fractions comprising, four bran fractions and six flour fractions, the latter representing three break fractions and three reductions. Determination of the ash contents of these fractions indicated that Break 1 and Reduction 1 were the purest (0.3% ash), followed by Break 2 and Reduction 2 (0.4%) and Break 3 and Reduction 3 (0.8 and 0.6%, respectively). Since the ash is derived from contamination with bran, it is likely that fractions 1 correspond to the central starchy endosperm and fractions 3 from the outer part. The range of variation in lipid composition within the two sample sets was similar, but multivariate analysis of the two datasets showed no clear correspondence between the fractions (Fig. 6). Most of the pearling fractions clustered together, with only the core clustering fairly close to the white flour fractions. It can therefore be concluded that pearling can be used to identify differences in the spatial distribution of components, but not to predict the compositions of fractions produced by roller milling, which require instead direct analysis.

Nevertheless, this study, and other comparisons of the compositions of mill streams (Prabhasankar et al., 2000; Nyström et al., 2007; Ramseyer et al., 2011), suggest that millers can indeed exploit differences in composition to produce specialist flours. For example, flour fractions from the central starchy endosperm cells should give highly elastic doughs suitable for breadmaking processes requiring high dough strength, such as the Chorleywood Breadmaking Process, while fractions from the outer layers should give more extensible doughs suitable for other products such as biscuits. Fractions from the outer layers may also have sufficient extensibility and tenacity to be incorporated into pasta making dough for fresh pasta or dry “special pasta”. Finally, differences in amylose:amylopectin ratio and in protein composition may also be exploited to improve the “processability” of foods requiring frozen or chilled technology (such as chilled doughs, bake–at-home breads and frozen cookie doughs), by increasing texture resilience.

## Declaration of competing interest

The authors have no competing interests.
